# Supplementary material for: “It Doesn’t Cure, but It Protects”: COVID-19 Vaccines through the Eyes of Children and Their Parents
Source: Vaccines (Basel). 2023 Jul 31;11(8):1305. doi: 10.3390/vaccines11081305 (PMC10459681; doi:10.3390/vaccines11081305)
Supplement: Supplementary file 1 [file vaccines-11-01305-s001.zip › vaccines-2457104-File S1.pdf]

Theme	Additional exemplary extracts
**Parents' perceptions and potential uptake of COVID-19 vaccines**	
**Optimism and hope**	Participant PC3-1: I was happy because many people have died. I know there were so many things said about the vaccine… but finally I said, "Oh! At least there is something that would save people" Some were saying vaccine would end COVID completely, but I don't think it is true.<br><br>Participant PC1-1: I was happy because it would minimize the risk of people contracting the virus provided people also do take care of themselves because this is no guarantee that when you have been vaccinated you would therefore not get infected with the virus. It is something that gives some hope when you have gotten the vaccination, like if you are using public transport, you would feel protected, and the virus will play far from you provided you also adhere<br><br>Participant PC1-3: For me it was a good thing for it to be made available because we have been using vaccines even in the past like for smallpox, chicken pox… we have been vaccinated before together with our children, so a vaccine is a positive thing that can prevent a problem that may later come
**Concerns and mistrust**	Participant PC1-3: Uh… I am just worried about the corrupt people who might manufacture fake vaccines and sell them to enrich themselves. So, we do have such people who are opportunists who would inject people with a fake thing. There is a lot of corruption in South Africa! And sometimes there could be people who would hijack vehicles that transport those vaccines before they can reach the masses locally. So, my worry is corruption in managing the vaccines.<br><br>Participant PC2-1: My hubby has co-morbidities, but he is allergic to shellfish. So, we are not 100% sure how the vaccines would be a fatality.
**Side effects**	Participant PS3-1: My concern is when will the vaccine reach us the community people? […] And how will people react to it physically and is it the real vaccine that people are waiting for?<br><br>Participant PC1-1: Uhm… my worry is that there are always aftereffects when you have taken any injection. I remember I once got a flu injection, but I still got symptoms of flu afterwards, the feeling was weird. I felt like I had flu. So, my worry is about the aftereffects of that vaccine that I might have. Another thing is that we have not heard anyone that has taken the vaccine and how they felt but if we could hear them saying they felt fine after taking the vaccine then our minds will relax.
**No concerns**	Participant PCH2-1: No, but I would not know. I have not seen anyone in the community that I know and how it affects them.<br><br>Participant PST3-1: I have no worry, I only have hope and, I am positive.<br><br>Participant PST1-2: For now, I have not heard anything, but we do not know the side effects because we have not heard anything.

	Participant PA2-1: At the moment, no (I do not have any worries) and that is why I am at ease with it.
**Parents' perspectives on COVID-19 vaccination for children**	
**Optimistic**	"Yes, if they want to (take a vaccine) but I would advise to take it for their safety." (PST3-1)<br><br>"Yes, when the opportunity comes, they (children) will (take the vaccine). Even if they are not sure I tell them that if they take it, they will be doing it for our family." (PA2-1)
**Sceptical**	"Honestly, I would take it first to see my reaction and then I can give it to my son so that I can know." (PO3-2)<br><br>"For now, there is no one who is going to take it until I understand what it does. I cannot say I want to be on trial- even the President and Minister said it is a trial! That is what they do so, it can either be positive or negative and you cannot compliment someone for driving a car drunk even vaccine is like a drunk person driving a car not knowing whether they will arrive safely or not" (PA3-1)
**Children's perceptions and potential uptake of COVID-19 vaccines**	
**Optimism and hope**	Participant PC1-4_F_17: It is coming to reduce the rate at which a number of people get infected. It is going to help us […] People will get it and the nation will get cured and protected.<br><br>Participant PCH1-2_M_17: It will protect us, and I heard a lot have been vaccinated around Gauteng. People have to get it! […] I am happy that I will not hear of people having symptoms […].<br><br>Participant PC1-2_M_11: It is that it would help by preventing the infection.
