# Supplementary material for: “It Doesn’t Cure, but It Protects”: COVID-19 Vaccines through the Eyes of Children and Their Parents
Source: Vaccines (Basel). 2023 Jul 31;11(8):1305. doi: 10.3390/vaccines11081305 (PMC10459681; doi:10.3390/vaccines11081305)
Supplement: Supplementary file 1 [file vaccines-11-01305-s001.zip › vaccines-2457104-File S2.pdf]

Table S1 Consolidated criteria for reporting qualitative studies (COREQ): 32-item checklist

COREQ items		Authors' notes and directions of content in paper
Domain 1: Research team and reflexivity		
Personal Characteristics		
1. Interviewer/facilitator	Which author/s conducted the interview or focus group?	Discussed under section 2 Materials and Methods in paragraph 4
2. Credentials	What were the researcher's credentials? E.g. PhD, MD	PhD
3. Occupation	What was their occupation at the time of the study?	Chief Research Specialist
4. Gender	Was the researcher male or female?	Female participants were interviewed by a female interviewer and male participants by a male interviewer.
5. Experience and training	What experience or training did the researcher have?	The study PI has more than 15 years research experience. All interviewers had a minimum of three years' experience in qualitative data collection, including telephonic data collection through our sister study 'Life during lockdown'. This is documented under section 2.1 Establishing trustworthiness of data.
Relationship with participants		
6. Relationship established	Was a relationship established prior to study commencement?	Yes, some participants were recruited from a sister study called Life During Lockdown and thus rapport was established through this study. For new participants, the interviewers built rapport throughout the study. Please see section 2.1 Estabilshing trustworthiness of data for further rdetails
7. Participant knowledge of the interviewer	What did the participants know about the researcher? e.g. personal goals, reasons for doing the research	Participants were informed of the study during the consenting

8. Interviewer characteristics	What characteristics were reported about the interviewer/facilitator? e.g. Bias, assumptions, reasons and interests in the research topic	process and were provided with full details on the study through the information sheet. See S1.
Domain 2: study design		
Theoretical framework		
9. Methodological orientation and Theory	What methodological orientation was stated to underpin the study? e.g. grounded theory, discourse analysis, ethnography, phenomenology, content analysis	This has been documented in the paper. Please see the methodological orientation of the study is reported under section 2 Materials and Methods
Participant selection		
10. Sampling	How were participants selected? e.g. purposive, convenience, consecutive, snowball	
11. Method of approach	How were participants approached? e.g. face-to-face, telephone, mail, email	
12. Sample size	How many participants were in the study?	
13. Non-participation	How many people refused to participate or dropped out? Reasons?	
Setting		
14. Setting of data collection	Where was the data collected? e.g. home, clinic, workplace	
15. Presence of non-participants	Was anyone else present besides the participants and researchers?	
16. Description of sample	What are the important characteristics of the sample? e.g. demographic data, date	
Data collection		
17. Interview guide	Were questions, prompts, guides provided by the authors? Was it pilot tested?	This has been documented in the paper. Please see section 2 Materials and methods, paragraph 4.
18. Repeat interviews	Were repeat interviews carried out? If yes, how many?	Yes, 3 formal interviews were conducted. However, the current paper draws on the data gathered via the first interview.
19. Audio/visual recording	Did the research use audio or visual recording to collect the data?	This has been documented in the paper. Please refer to section 2 Materials and methods and section 2.1 Establishing trustworthiness of data.
20. Field notes	Were field notes made during and/or after the interview or focus group?	
21. Duration	What was the duration of the interviews or focus group?	
22. Data saturation	Was data saturation discussed?	

23. Transcripts returned	Were transcripts returned to participants for comment and/or correction?	
Domain 3: analysis and findingsz		
Data analysis		
24. Number of data coders	How many data coders coded the data?	
25. Description of the coding tree	Did authors provide a description of the coding tree?	
26. Derivation of themes	Were themes identified in advance or derived from the data?	
27. Software	What software, if applicable, was used to manage the data?	
28. Participant checking	Did participants provide feedback on the findings?	
Reporting		
29. Quotations presented	Were participant quotations presented to illustrate the themes / findings? Was each quotation identified? e.g. participant number	Yes, please see section 3 Results along with S1 for additional extracts.
30. Data and findings consistent	Was there consistency between the data presented and the findings?	Yes. This is discussed in the paper. Please see section 3 Results.
31. Clarity of major themes	Were major themes clearly presented in the findings?	
32. Clarity of minor themes	Is there a description of diverse cases or discussion of minor themes?	

Adapted from: [17]Tong A, Sainsbury P, Craig J. Consolidated criteria for reporting qualitative research (COREQ): a 32-item checklist for inter-views and focus groups. International journal for quality in health care. 2007 Dec 1;19(6):349-57. https://doi.org/10.1093/intqhc/mzm042
