# Supplementary material for: “It Doesn’t Cure, but It Protects”: COVID-19 Vaccines through the Eyes of Children and Their Parents
Source: Vaccines (Basel). 2023 Jul 31;11(8):1305. doi: 10.3390/vaccines11081305 (PMC10459681; doi:10.3390/vaccines11081305)
Supplement: Supplementary file 1 [file vaccines-11-01305-s001.zip › vaccines-2457104-File S3.pdf]

Interview guide: Timepoint 1

Children and teens

Introduction

Thank you for taking the time to do this interview with me. For this interview we just want to catch up with you to see you and your family are doing. Remember there are no wrong or right answers. This is just for us to understand how you are doing, so try to be as honest as possible.

General

Before we start with some of the more formal questions, I thought it would be good for us to get to know each other a little. So, can you maybe tell me a little bit about yourself?

- If you could be any animal, which would you choose and why?
- How would you describe a perfect day?
- What is your favorite food and why?
- How has the past week been for you and your family?
  - Did anything happen that made you feel happy?
  - Did anything happen that made you feel sad?

Thoughts on COVID-19

We are now going to ask you some questions about COVID-19 or the coronavirus.

- Let's say your friend does not know what COVID-19 or the coronavirus is. What would you say to help them understand what it is?

- Where do you normally get your information about the COVID-19?
  - Where did you hear about COVID-19 or the coronavirus for the first time?

- How do you feel about COVID-19 or the coronavirus?
  - Do you think it 'real'? (there are people who think it is all a myth or not true)
  - What are some of the things that you have heard your friends say about COVID-19 or the coronavirus?
    - Do you believe them?
- Do you know if anyone in your family has tested positive for COVID?
  - If yes: How did you feel when you found out?
    - Where you worried about anything or scared?
      - If yes: How did you make yourself feel better?

- Have you ever felt worried that you had COVID-19?
  - If yes: Why did you think you might have COVID-19 or the coronavirus

        - Which symptoms did you have?
        - How long did you feel sick for?
        - Do you feel fully recovered now?
        - Did you go for a test?
        - If no test: What was the reason you did not go for a test?
        - If you were to have COVID like symptoms in future, would you go for a test? Why / why not

- What do you think is a good treatment for COVID-19 or the coronavirus?
    - Are there any traditional/cultural or religious 'treatments' that people are using in your community?

- Have you heard that we will be getting the COVID-19 vaccines?
    - What do you think this vaccine will do?
        - Is there anything about the vaccines that make you scared? Please explain
        - Is there anything about the vaccines that make you happy? Please explain
    - Who told you about the vaccines?
    - Will you take the vaccine? (please explain your answer)

About the regulations

As you know, the government put in place various rules and lockdown levels to protect South Africans during the pandemic.

- How do you feel about the lockdown?
    - Do you think it is a good thing or a bad thing? Why?
    - Which of the lockdown rules do you think are the most important?
    - Which of the lockdown rules do you like the most?
        - Which do you hate?

For adolescents: One of the regulations that caused a lot of conversations is the alcohol ban.

- What are your thoughts about the alcohol ban?
- Do you agree with this ban? Please tell us why/ why not
- Do you think there has been any negative outcomes associated with the alcohol ban? (has it caused any problems?) Please elaborate
- Do you think there has been any positive outcomes associated with the alcohol ban? Please elaborate
- Do you know of anyone in your community or perhaps one of your friends that were able to buy alcohol during the ban? What can you tell us about their experience and the cost of the alcohol?

About family life

We are now going to talk your family.

    - Who would you say are part of your family?
    - What has it been like to be with your family now that the COVID pandemic is happening?
        - Are there things that you do more of together as a family? Can you tell me about this?
        - Are there things that you do less of as a family? Can you tell me about this?
        - What are the things that you do as a family that make you feel happy?
        - What are the things that you do as a family that make you feel sad?
    - What do you think are some of the challenges or problems that your family has faced over the past year of the lockdown?
    - Can you tell me about things that you and your family do to distract yourselves from the challenges or worries during the lockdown?

- Do you think the lockdown, or the pandemic had any impact on the family in terms of finances? Please elaborate

- How do you think COVID-19, or the lockdown has affected your romantic relationship or the possibility of having a romantic relationship? Please explain

- How do you think COVID-19, or the lockdown has affected your friendships? Please explain

Coping and support needs (general)

We now want to talk about your life more generally.

- What have you been doing to make yourself feel better during the lockdown?
- What have you been doing to make yourself feel better when you feel worried about the coronavirus?
- Who has been helping you to feel better when you are scared or feel worried about the coronavirus?
    - What would they say or do to make you feel better?
- How do you think others in your family feel about the coronavirus?
-

Ending the interview

We have come to end of our first interview.

- How do you feel about the questions I asked you?
- Were any of the questions difficult to answer? Which ones?
- How are you feeling now that the interview is over?
- Do you have any questions for me?

Interview guide: Timepoint 1

New adult participants

Introduction

Thank you for taking the time to do this interview with me. For this interview we just want to catch up with you to see you and your family are doing. Remember there are no wrong or right answers. This is just for us to understand how you are doing, so try to be as honest as possible.

General

Before we start with some of the more formal questions, I thought it would be good for us to get to know each other a little. So, can you maybe tell me a little bit about yourself?

- For examples: what you do, studies (for students, adolescents and children), your home life
- How would you describe your personality?
- How would you describe your perfect day?
- How has the past week been for you and your family?
    - Was there anything you struggled with? (please elaborate)
    - Was there anything that you were happy about? (please elaborate)

Thoughts on COVID-19

As you know, this study is about understanding how the COVID-19 pandemic influences families. So, our first couple of questions will focus on your perspectives on COVID-19

- How would you describe the COVID-19 pandemic to someone who has not heard about it? (please elaborate)
- Where do you generally get your information about the COVID-19?
    - Check whether they use online platforms like Facebook, Twitter or Instagram, OR government websites OR WhatsApp chatline etc
- How do you feel about COVID-19?
    - Do you think it 'real'? (there are people who think it is all a myth or not true)
    - What are some of the information about the pandemic that you have received that you thought was not true OR that you did not believe?

- Do you know of anyone in your family that has tested positive for COVID?
    - Can you tell me about this experience?
    - How are they doing now?
    - Have you ever felt symptoms of COVID but did not test?
        - What was the reason you did not go for a test?
        - Which symptoms did you have?
        - How long did your symptoms last?

        - Do you feel fully recovered now?
        - If you were to have COVID like symptoms in future, would you go for a test? Why / why not
- What do you know about treatment for COVID-19?
    - Are there any traditional/cultural or religious 'treatments' that people are using in your community? (home remedies)

- What are your thoughts about the COVID-19 vaccines?
    - How did you feel when you found out that vaccines will be made available?
    - How did you find out about the vaccines?
    - Will you take the vaccine? (please explain your answer)
    - Would you let you child/ren take the vaccine? (please explain your answer)
    - What are your worries or concerns related to the vaccine?
    - What are you happy about related to the vaccine?

About the regulations

As you know, the government imposed various regulations and lockdown levels to protect South Africans during the pandemic.

- How do you feel about the different lockdown levels that have been imposed?
- Which lockdown level did you struggle with the most?
- Thinking about the various regulations, which do you think are the most important ones for people to uphold?
    - Which of the regulations bother you the most and why? (or do you struggle with the most)
- The lockdown and the regulations come with various restrictions. How would you describe the impact of these on your daily life? (made it easier or more difficult? Please elaborate)
    - What were some of the main challenges that you faced? (visiting family, funerals, accessing food or supplies, traveling to work etc.)

- One of the regulations that caused a lot of conversations is the alcohol ban.
    - What are your thoughts about the alcohol ban?
    - Do you agree with this ban? Please tell us why/ why not
    - Do you think there has been any negative outcomes associated with the alcohol ban? (has it caused any problems?) Please elaborate
    - Do you think there has been any positive outcomes associated with the alcohol ban? Please elaborate
    - During the ban, were you able to buy alcohol at all?
        - If yes, where were you able to buy this and what were the costs?
    - Do you know of anyone else in your community or perhaps one of your friends that were able to buy alcohol during the ban? What can you tell us about their experience and the cost of the alcohol?

About family life

I would now like to talk to you about your family.

- How has life been for you as a family during lockdown?
- What are some of the things that you have done **more of** over the past year of the lockdown?
  - How do these things make you feel (are you happy, sad, angry, worried etc. about it? Please elaborate)
- What are some of the things that you do less as a family over the past year of the lockdown?
  - How do these things make you feel (are you happy, sad, angry, worried etc. about it? Please elaborate)
- What are some of the challenges that you, as a family, have faced over the past year of the lockdown?
  - Were any of these challenges worse during a particular lockdown level? Please explain
  - Have you felt any improvements?
- Can you tell me about things that you and your family do to distract yourselves from the challenges or worries during the lockdown?

[living with spouse/married/ in a relationship] How do you think the pandemic and lockdown has affected your relationship with your spouse? Please explain?

- Has your relationship changed?
  - For better or is it more challenging? Please explain

- Has the lockdown or the pandemic had any impact on the family in terms of finances? Please elaborate

Coping and support needs (general)

- What have you generally been doing to cope or deal with the challenges you have faced because of the pandemic or lockdown? Please elaborate
- What do you think you need support with?
- Have you reached out to anyone (friends, family, formal support like therapist, pastor, doctor) to get support for yourself?
- How are others in your family coping with the pandemic and the lockdown?
  - Do you think they need support?
  - What kind of support do you think they need?

Ending the interview

We have come to end of our first interview.

- How do you feel about the questions I asked you?

- Were any of the questions difficult to answer? Which ones?
- How are you feeling now that the interview is over?
- Do you have any questions for me?
